# 1505. Clinical characterization of TB/HIV naive coinfection patients enrolled in a TB program in Cali - Colombia.

**DOI:** 10.1093/ofid/ofad500.1340

**Published:** 2023-11-27

**Authors:** Jenny Patricia Muñoz-Lombo, Julio Llanos-Torres, David E Rebellon-Sanchez, Sofía Alexandra Montes-Tello, José Fernando García-Goez, Fernando Rosso

**Affiliations:** Fundacion Valle del Lili, Cali, Valle del Cauca, Colombia; Fundación Valle del Lili, Cali, Valle del Cauca, Colombia; Fundacion Valle del Lili, Cali, Valle del Cauca, Colombia; Fundación Valle del Lili, Cali, Valle del Cauca, Colombia; Fundación Valle del Lili, Cali, Valle del Cauca, Colombia; Fundación Valle del Lili, Cali, Valle del Cauca, Colombia

## Abstract

**Background:**

Although antiretroviral therapy (ART) is beneficial for patients with TB/HIV coinfection, there are issues associated with concomitant therapy administration such as overlapping drug toxicity profiles, drug interactions, and the possibility of developing immune reconstitution inflammatory syndrome (IRIS). The clinical experience in managing patients with TB who develop de novo HIV is poorly described in Colombia and the prevalence of IRIS in our context is unknown. This study aims to describe the clinical characteristics of patients with TB who develop de novo HIV coinfection and determine the prevalence of IRIS following treatment initiation.

**Methods:**

Retrospective, observational study. Patients in the TB program whit de novo HIV coinfection were included.

**Results:**

A total of 39 patients were included. Most of the patients were male (64%) and of mixed race (58%) with an urban origin (92%). Smoking was documented in 24% of the patients and the Bacille Calmette-Guérin (BCG) TB vaccine was recorded in 48% of the patients. Pulmonary TB occurred in 74% of the patients, while miliary TB and lymph node involvement occurred in 26%. Meningeal TB and CNS TB were present in 15% of the patients, peritoneal TB in 18%, and pleural TB in 5.1%. More than 70% of the patients experienced weight loss and dyspnea (74% and 76%, respectively). Antiretroviral treatment was administered to 46% of the patients with EFV-AZT/3TC, to 21% with LPV/R+ABC/3TC, and to 14% with EFV+TDF/FTC. Individualized tuberculosis treatment was administered to 38.48% of the patients. Of the patients, 19% (5/27) developed IRIS, of which three developed TB-IRIS. The mortality rate was 2.6%, while the cure rate was 18%.

**Conclusion:**

our study sheds light on the clinical characteristics of patients with TB who develop de novo HIV coinfection in Colombia. The high prevalence of smoking and low prevalence of BCG vaccination among these patients are notable findings. Additionally, our study highlights the importance of individualized treatment for TB and the need for careful management of patients receiving ART to minimize the risk of developing IRIS. Further research is warranted to explore the prevalence of IRIS in our context and to develop strategies to reduce its incidence.

**Disclosures:**

**All Authors**: No reported disclosures

